# Tracking Potentiating States of Dissociation: An Intensive Clinical Case Study of Sleep, Daydreaming, Mood, and Depersonalization/Derealization

**DOI:** 10.3389/fpsyg.2016.01231

**Published:** 2016-08-17

**Authors:** Giulia L. Poerio, Stephen Kellett, Peter Totterdell

**Affiliations:** ^1^Department of Psychology, University of YorkYork, UK; ^2^Department of Psychology, University of SheffieldSheffield, UK; ^3^Centre for Psychological Services Research, University of Sheffield and Sheffield Health and Social Care NHS Foundation TrustSheffield, UK

**Keywords:** daydreaming, mindwandering, dissociation, depersonalization, sleep, emotion, experience-sampling methodology, clinical case study

## Abstract

This study examined in real time the role of sleep and daydreaming as potentiating states for subsequent dissociation in depersonalization/derealization disorder (DDD). Research and theory suggests that dissociation may be exacerbated and maintained by a labile sleep-wake cycle in which “dream-like” mentation intrudes into waking life and fuels dissociative symptoms. We explore and extend this idea by examining the state of daydreaming in dissociation. Daydreaming is a state of consciousness between dreaming and waking cognition that involves stimulus-independent and task-unrelated mentation. We report the results of a unique intensive *N* = 1 study with an individual meeting diagnostic criteria for DDD. Using experience-sampling methodology, the participant rated (six times daily for 40 days) current daydreaming, mood, and dissociative symptoms. At the start of each day sleep quality and duration was also rated. Daydreaming was reported on 45% of occasions and significantly predicted greater dissociation, in particular when daydreams were repetitive and negative (but not fanciful) in content. These relationships were mediated by feelings of depression and anxiety. Sleep quality but not duration was a negative predictor of daily dissociation and also negatively predicted depression but not anxiety. Findings offer initial evidence that the occurrence and content of daydreams may act as potentiating states for heightened, in the moment, dissociation. The treatment implications of targeting sleep and daydreaming for dissociative disorders are discussed.

## Introduction

Detaching from one's immediate surroundings when engrossed in an exhilarating novel or experiencing the energized focus of “flow” at work, are examples of dissociative experiences that can occur in everyday life. Although, typically viewed on a continuum, clinical forms of dissociation are not simply reflective of psychological absorption. Dissociation in dissociative disorders typically involves substantial ongoing problems in integrating thoughts and feelings into consciousness and memory, with associated poor psychosocial functioning (Waller et al., 1996). Prevalence estimates for dissociative disorders range from 4 to 29% of the population and typically involve two common aspects of dissociation: depersonalization (i.e., feelings of disconnection from one's self such as feeling like a robot or automaton) and derealization (i.e., feeling disconnected from ongoing reality, as if the world is distorted or moving in slow motion; van der Kloet et al., 2012b). Recent research and theory proposes that dissociative disorders are maintained and exacerbated by a labile sleep-wake cycle. In this cycle, imaginative, “dream-like,” mentation intrudes into waking life, which, in turn, contributes to dissociative experiences and symptoms. In this paper, we present an initial test and extension of this theory by examining the role of daydreaming in dissociation. Specifically, we view daydreaming as a form of dream-like mentation and examine its relationship with sleep, mood, and dissociative symptoms in a unique experience-sampling study with an individual meeting diagnostic criteria for depersonalization/derealization disorder (DDD; American Psychiatric Association, 2013).

### Dissociation and a labile sleep-wake cycle

The etiology of dissociative disorders has historically been proposed to reflect either coping with early childhood adversity/trauma (e.g., Sanders and Giolas, 1991; Gleaves, 1996; Bremner, 2010) or social learning/expectancies (e.g., Lilienfeld et al., 1999). However, more contemporary approaches (e.g., Watson, 2001; Lynn et al., 2012; van der Kloet et al., 2012b; van Heugten–van der Kloet et al., 2014) highlight the important role of sleep experiences for the proximal development and severity of subsequent dissociation. According to this model “sleep-related deficiencies in cognitive control may promote an influx of imaginative, dreamlike mentation in daily life that contributes to dissociative symptoms such as depersonalization and derealization” (van der Kloet et al., 2012b, p. 167). A labile sleep-wake cycle is proposed to promote dissociation by “pushing” sleep-like mentation into waking consciousness, which then fuels fantasy-proneness, and is associated with cognitive failures, and feelings of depersonalization/derealization.

Several studies support the close association between sleep disturbance and dissociative symptoms. Correlational research consistently shows that sleep disturbances (e.g., unusual sleep experiences) are positively correlated with dissociation (e.g., Watson, 2001; Levin and Fireman, 2002; Agargun et al., 2003; Giesbrecht and Merckelbach, 2004; Koffel and Watson, 2009a) and experimental studies demonstrate that dissociative symptoms are heightened by sleep-wake cycle disruptions (Giesbrecht et al., 2007; van Heugten–van der Kloet et al., 2015). Although, this research indicates that sleep disturbances may exacerbate dissociation, research has yet to fully identify and examine the “dream-like mentation” purported to precede and fuel dissociation in daily life. Research has therefore typically focused on the distal relationship between nighttime experiences and daytime dissociation rather than examining how different states of consciousness may be linked to current dissociation as they actually occur during wakefulness. We address this gap by examining the state of daydreaming in dissociation, which can be conceptualized as a state of consciousness in between fully-focused waking thought and sleep mentation (i.e., dreaming). We draw on existing research and theory on daydreaming and emotion to extend theoretical ideas about dream-like mentation (daydreaming) as a potentiating state for heightened dissociation. To ensure external validity and clinical credibility, the theory is tested in an experience-sampling study with an individual meeting diagnostic criteria for DDD (American Psychiatric Association, 2013).

### Daydreaming as dream-like mentation

Daydreaming (also variously referred to as mindwandering, spontaneous thought, off-task thinking, stimulus-independent thought) can be defined as mental content that is both stimulus-independent and task-unrelated (Stawarczyk et al., 2011). Daydreaming is stimulus-independent because its content is not directly related to the processing of the immediate environment (i.e., it is internally generated) and it is task-unrelated because its content is unrelated to the progression or completion of the current goal(s) in the external environment. Thus defined, daydreaming is estimated to occupy between a third and a half of waking life (Klinger and Cox, 1987-1988; Killingsworth and Gilbert, 2010), during which thought operates in a more free-flowing, diffuse, and less directed manner than during other kinds of waking mentation (e.g., the deliberate and fully focused thought involved in calculating one's monthly finances; Klinger, 2009).

Several researchers have noted parallels between the daydreaming state and dreaming/sleeping states (e.g., Raichle, 2009; Christoff et al., 2011; Fox et al., 2013; Klinger, 2013; Wamsley, 2013), supporting the notion that daydreaming lies in the middle of the sleep-wake mentation continuum (e.g., Hartmann, 2010; Montangero, 2012). Daydreams show substantial similarities with dreams both in terms of their content and neurophysiological basis (see Fox et al., 2013; Wamsley, 2013, for reviews). Both daydreaming and dreaming involve activation of the default-mode network, which is a core network of regions including the medial prefrontal and cingulate cortex, the medial temporal lobe, the lateral parietal cortex, and areas of the cerebellum and striatum (Buckner et al., 2008). Domhoff and Fox (2015) have recently conceptualized dreaming as an intensified form of daydreaming because of the increased activation in areas of the default mode network that support sensorimotor imagery in REM sleep relative to the “resting” states characteristic of daydreaming. These authors further suggest that “people can indeed drift into dreaming during periods of relaxed wakefulness and mindwandering” (p. 349) which mirrors suggestions that “dream-like” or even dreaming mentation can enter consciousness during wakefulness through daydreaming.

**Figure 1:**
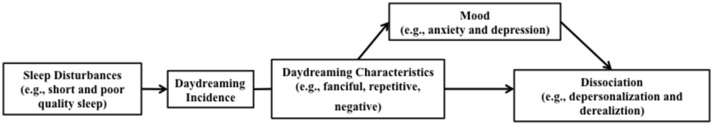
**A proposed model of how daydreaming is related to dissociation**.

If daydreaming can be one way in which dream-like mentation intrudes into consciousness in dissociative disorders and exacerbates symptoms, then how might these relationships present and unfold in daily life? We propose an initial model of these relationships (shown in Figure 1) in which sleep disturbances exacerbate/increase daydreaming; and, in turn, daydreaming (and its characteristics) elicits negative mood and subsequent dissociation. We now review the evidence for each component of this suggested model within existing literature.

### Sleep disturbance and daydreaming frequency

Several lines of converging evidence support the proposal that, at least in normative samples, sleep disturbances are related to increased mindwandering/daydreaming. In both laboratory settings and in daily life, daytime fatigue, and sleepiness have been consistently associated with increased momentary mindwandering and daydreaming (e.g., Antrobus et al., 1966, 1967; Manly et al., 2002; McVay and Kane, 2009). Sleep duration has been negatively associated with hours spent daydreaming (Kunzendorf et al., 1983) and sleep deprivation has also been associated with higher rates of later daydreaming (Mikulincer et al., 1990). More recently, daydreaming frequency has been positively associated with various aspects of poor sleep quality (such as sleep latency and disturbances; Carciofo et al., 2014). People who report poorer sleep quality and more unusual sleep experiences (e.g., sleep paralysis and lucid dreaming) also report daydreaming more frequently (Denis and Poerio, 2016).

There are several possibilities for how sleep disturbances might predict the incidence of daydreaming in dissociative disorders. First, as implied by models of dissociation (van der Kloet et al., 2012b), poorer sleep could make the intrusion of dream-like mentation via daydreams more common in dissociation. Second, poorer sleep quality could increase levels of fatigue or reduce metacognitive control which might then be associated with greater daydreaming, in turn increasing the likelihood of a dissociative experience in someone prone to dissociation. Indeed previous research has linked dissociation with increased distractibility and attentional difficulties (Guralnik et al., 2007). Another possibility is that both the labile sleep-wake cycle and daydreaming incidence may be underlined by a latent trait in dissociation characterized by attentional/metacognitive control difficulties (which in itself could make intrusions of sleep experiences into waking thought more likely). Although, we do not test specifically test these possible mechanisms in the present study, we examine for the first time whether the basic association between sleep disturbances and increased daydreaming found in non-clinical samples also occurs in dissociative disorder. Based on the reviewed evidence, we predict that measures indexing sleep disturbance (e.g., sleep duration and quality) would also be associated greater daydreaming incidence in dissociative disorders.

### Daydreaming and dissociative symptoms

Daydreaming is typically conceived of as state or symptom of both normative (e.g., Butler, 2006) and pathological dissociation (Holmes et al., 2005; Lynn et al., 2012). Indeed, measures of dissociation typically include items related to psychological absorption/daydreaming (e.g., “becoming so involved in a fantasy or daydream that it feels as though it were really happening to you” from the Dissociative Experiences Scale; Carlson and Putnam, 1993). Additionally, cross-sectional research has shown that daydreaming styles are positively associated with both dissociative experiences (Segal and Lynn, 1993) and clinical dissociation (Levin and Spei, 2004). More recent research using a large sample has associated the tendency to daydream more frequently with having more dissociative experiences (Denis and Poerio, 2016). However, the existing research has treated daydreaming as a trait or global variable (e.g., daydreaming style or typical frequency) and has not yet examined whether the state of daydreaming is associated with dissociation. This makes it difficult to ascertain whether daydreaming is a concomitant of dissociation or, as theories might suggest, a state that precedes and fuels dissociative symptoms. Whether momentary daydreams are associated with symptoms of dissociation is therefore an open question. We suggest that rather than daydreams *per se* being associated with worse dissociation, the effect of momentary daydreams on dissociative symptoms will depend on the characteristics of specific daydreams and their relationship with affective states.

### Daydreaming characteristics, mood, and dissociation

Although, daydreaming has been previously labeled as a homogeneous experience that has negative effects on emotional well-being (e.g., Killingsworth and Gilbert, 2010), emerging research has consistently supported the view that daydreaming is a heterogeneous experience and that it is through this heterogeneity that certain costs and benefits of the experience emerge (Smallwood and Andrews-Hanna, 2013). With respect to emotional well-being, a number of studies indicate that the characteristics of daydreams (e.g., what people daydream about) determine whether daydreaming has a positive or negative effect on emotional experiences. For example, research has consistently found that daydreams with a positive emotional and social content are associated with beneficial affective outcomes (e.g., greater feelings of happiness, reduced loneliness; Poerio et al., 2013, 2015a,b, 2016). Analogously, other research has identified the specific characteristics of daydreaming related to negative affective outcomes. In particular, repetitive, self-focused, unintentional, and negative daydreams have been linked with poorer emotional well-being and psychological disorder (Ottaviani and Couyoumdjian, 2013; Deng et al., 2014; Marchetti et al., 2014, 2016). This suggests that although daydreaming is likely to be associated with affective outcomes and psychopathological symptomology, this relationship will likely depend on the characteristics of daydreaming.

Drawing on this research and the importance of viewing daydreaming as a heterogeneous experience, we sought to capture pertinent characteristics of daydreaming and their links to mood and dissociation in the present study. Specifically, we measured the emotional valence, repetitive, and fanciful nature of individual daydreams. The first two characteristics were chosen because research in both daydreaming and repetitive thought has consistently associated negative and repetitive thoughts with the occurrence and maintenance of psychopathology (e.g., Segerstrom et al., 2003; Watkins, 2008). In light of this evidence, we expected that daydreams that were more negative in valence and repetitive would be associated with greater dissociation. The fanciful nature of daydreams was chosen as a characteristic of specific clinical relevance to dissociative disorders. Fantasy proneness (i.e., the tendency to engage in vivid imaginative experiences) is a consistent correlate of dissociation in both clinical and non-clinical samples (e.g., Rauschenberger and Lynn, 1995; Giesbrecht and Merckelbach, 2006). Indeed, fantasy proneness is a personality trait that is proposed to map onto dissociation, and fantasy intrusions into waking states are proposed to be a symptomatic and maintenance factor for dissociative disorders (van der Kloet et al., 2012b). To our knowledge, no previous research has examined fantasy as a current state (e.g., in terms of on-going fanciful daydreams) to assess whether such fanciful cognition is associated with dissociation. Based on previous research and theory, we expected more fanciful daydreams to be associated with greater dissociation.

### The present study

Building on existing theories of sleep disturbances in dissociation and research on daydreaming, we propose an initial model of how sleep and daydreaming interrelate to predict negative mood and dissociative symptoms during dissociative disorder. Specifically, we predict that sleep disturbances will be associated with a greater incidence of daydreaming; and that daydreaming will be associated with greater symptoms of dissociation and negative mood depending on the nature of those daydreams (i.e., the extent to which they are fanciful, repetitive and negative). We tested this model by sampling daydreaming episodes, sleep experience, negative mood (anxiety and depression), and dissociative symptoms of an individual with DDD in an intensive single-case experience-sampling study. Intensive quantitative single clinical case study research has a long and significant heritage and is particularly indicated in the “hourglass model” (Salkovskis, 1995) when there is a lack of evidence for clinical phenomena and a need for associated theory building. Experience-sampling involves reporting on targeted momentary experiences on each occasion participants are signaled over a period of time (Stone et al., 1991). In a clinical context, experience-sampling enables an examination of how fluctuations in everyday experience (e.g., daydreaming) relate to changes in clinical symptoms (e.g., dissociation) within a patient over time, which can be different from between person relationships (Tennen and Affleck, 2002). This method has been found to be particularly useful in *N* = 1 outcome studies (e.g., Totterdell et al., 2012), because it has the advantage of capturing relationships between, and change in, clinical symptoms much closer to their occurrence, compared to traditional retrospective nomothetic outcome measures.

## Materials and methods

### Participant

The participant was a 24 years old white-British male. The participant had a history of childhood trauma (poor attachment and an assault) and associated attendance in child and adult psychiatric services. Previous psychiatric assessment on three separate occasions had diagnosed a dissociative disorder, with childhood onset. The participant had also been previously diagnosed with Generalized Anxiety Disorder as a child by a psychiatrist. There were no previous episodes of psychiatric admission to an in-patient setting. The participant approached the research team volunteering to participate in research because of his diagnosis and the impact he recognized the dissociation had on his ability to function. Throughout the duration of the study, the patient was taking a low dose of an anti-convulsant and this did not change. Prior to the current study the patient underwent psychological assessment in the form of the (a) Structured Clinical Interview for DSM-IV Dissociative Disorders (SCID-D; Steinberg, 1993) and (b) Clinician Administered Dissociative States Scale (CADSS; Bremner et al., 1998). The SCID-D findings were that the patient met diagnostic criteria for DDD (American Psychiatric Association, 2013) and, on the CADSS, the participant scored 74, which is above the mean for dissociative disorder (Bremner et al., 1998). In brief, the participant described chronic feeling of disconnection from his immediate environment, frequently occupying a cut-off dreamlike state and that he frequently experienced himself as an unreal, disembodied, robot-like figure. Regarding sleep, the participant stated at assessment that he was a vivid dreamer and that his sleep was chaotic and labile; he frequently went to bed much later than the average person (therefore sleeping later in the day) and often had disturbed and broken sleep.

### Experience-sampling protocol

A signal-contingent experience-sampling protocol (Wheeler and Reis, 1991) was used to obtain repeated data on dissociation, daydreaming, mood and sleep. The participant was signaled on a smartphone via text message six times daily for 40 days with a link to answer online questionnaires regarding dissociation, daydreaming, mood and sleep (see for example, Poerio et al., 2015b, 2016). The six signals were scheduled to occur in three pairs of two signals (separated by between 5 and 10 min) during the following time slots, which were chosen according to the participant's typical waking hours: 12:00-16:30, 16:30-21-30, 21:30-02:30. The first signal in each pair occurred at a random time within each time block with the constraint that pairs of consecutive signals were at least an hour apart. The pairing of signals in each time slot was originally designed to allow an examination of temporal contiguity (by splitting the data into two alternate time-series) over a longer period but the study was curtailed to 40 days due to the participant's new work commitments, so the reduced number of observations meant that this could not be examined.

### Procedure

After completing the psychological assessment, the participant met with the researchers on two occasions to discuss the nature of the study and what it would involve. In the first session, the experience-sampling design and appropriate times for signaling were negotiated to fit in with daily routines. We also collaborated with the participant regarding wording of items to ensure that measures of dissociative symptoms were grounded in the participant's daily experiences of derealization and depersonalization (Kellett and Beail, 1997). In the second session, the participant was provided with detailed instructions for completing the study. He was given a written and verbal description of daydreaming and his understanding of the concept was checked and discussed. In line with previous studies (e.g., Poerio et al., 2015b, 2016), a daydream was defined as a series of connected thoughts and/or images where that mental content is not about whatever mental or physical activity one is engaged in at the present moment. Next, the participant was provided with a demonstration of the text message with online questionnaire link and verbal explanation of the meaning and response of each questionnaire item. All instructions for how to complete the study were also provided in written format for later reference. Informed consent was obtained and a start date for the experience-sampling was agreed. At the end of the training session, the participant completed global measures indexing dissociative experiences over the past month. Ethical approval for this study was obtained from the University of Sheffield Psychology ethics committee and was conducted in line with British Psychological Society ethical guidelines.

### Global dissociation measures

**Cambridge Depersonalization Scale** (CDS; Sierra and Berrios, 2000). Twenty-nine-items measured the frequency and duration of depersonalization and derealization symptoms associated with depersonalization disorder including: abnormal sensory experiences (e.g., “*Familiar voices (including my own) sound remote and unreal*”), cognitive and emotional complaints (e.g., “*When I weep or laugh, I do not seem to feel any emotions at all*”) and space and time distortions (e.g. “*Objects around me seem to look smaller or further away*”). Each item was rated on two likert scales for frequency over the past month (1 = *never*, 5 = *all the time*) and duration of the experience (1 = *a few seconds*, 6 = *more than a week*). Average scores for frequency and duration were calculated with higher values indexing more frequent and longer-lasting symptoms of depersonalization over the preceding month.

**Dissociative Experiences Scale** (DES-II; Carlson and Putnam, 1993). Twenty-eight-items measured the frequency of dissociative experiences over the past month (e.g., “*Finding yourself in a place and having no idea how you got there*”). Each item was rated using 100-point sliding scales (higher values indicating greater frequency). Scores for each item were summed to create an overall score with higher scores indicative of greater dissociative experiences over the past month. The measure also included three subscales, each with 6-items, indexing amnesia (e.g., “*Finding yourself dressed in clothes that you don't remember putting on*”), depersonalization/derealization (e.g., “*Looking in the mirror and not recognizing yourself*”), and absorption (e.g., “*Sitting staring off into space, thinking of nothing, and not being aware of the passage of time*”).

### Experience-sampling measures

At each signal, the first question always asked about daydreaming and, if applicable, daydreaming characteristics. These questions were followed by items regarding mood and dissociative symptoms, and finally alcohol consumption within the past 3 h. For the first signal of every day, the daydreaming questions were followed by items indexing the previous night's sleep. The set of experience-sampling items was kept brief to minimize participant burden, in line with recommended practice (Bolger et al., 2003; Christensen et al., 2003).

#### Daydreaming incidence and characteristics

The participant was asked “*Right before you were signaled, or within the last 5 min, were you daydreaming?*” (0 = *No*, 1 = *Yes*). When the participant answered affirmatively, he was asked several other questions about the characteristics of that daydream. Each daydream was rated on three 7-point scales according to its fanciful nature (1 = *completely realistic*, 7 = *completely fanciful*), emotional valence (1 = *very negative*, 7 = *very positive*), and novelty (1 = very *repetitive*, 7 = *completely novel*). The order of these items was individually randomized for each presentation.

#### Current mood and dissociative symptoms

In response to the question “*How do you feel right now?*” the participant answered two items concerning mood that indexed anxiety (“*anxious*”) and depression (“*depressed*”), and seven items concerning dissociative symptoms that included three items for experiences of derealization (“*Cut off from the world around me*,” “*Detached from my surroundings*,” “*That the world around me seems to look smaller or larger*”), and four items for experiences of depersonalization (“*Emotionally numb*,” “*That I am outside of my body*,” “*That I am robotic*,” “*That I am a detached observer*”). The symptoms of dissociation were taken from the Cambridge Depersonalization Scale (CDS; Sierra and Berrios, 2000) and were adapted to effectively tap into the participant's own experience of dissociation. This “client centered” and idiographic measurement of clinical phenomena is at the methodological heart of *N* = 1 research (Totterdell et al., 2012). Detailed efforts were therefore made to ensure the high face validity of dissociative items with the participant, so that the items were grounded in their daily experience of dissociation. This is in keeping with good practice in the design of *N* = 1 research (Kellett and Beail, 1997). For example, CDS item 1 “out of the blue, I feel strange, as if were not real or as if I were cut from the world around me” was shortened in collaboration with the participant to 'cut off from the word around me' with the stem of I am currently feeling. The order of all these items was individually randomized for each presentation and items were answered on a 5-point scale from 1(*not at all*) to 5(*extremely*). The seven dissociative symptom items were averaged at each time point to create an overall score, where higher values indicated greater current experience of dissociation in general (derealization and depersonalization; α = 0.81).

#### Alcohol and medication

The participant indicated his recent alcohol consumption (“*Have you consumed any alcohol in the last 3 h?*”; 0 = *No, 1* = *Yes*) and, using a free text response box, whether there had been any deviations from his medication (of which there were none reported during the study). Alcohol consumption was measured to be included as a control variable in our analyses. This was because the participant indicated during assessment that alcohol typically increased his tendency to dissociate (although there was no evidence of alcohol dependency from the assessment). This is also consistent with previous research suggesting that clinical dissociation is made worse by alcohol consumption (Baker et al., 2003).

#### Sleep

The participant was asked to provide the previous night's time of sleep onset and wakening (“*What time did you go to sleep/wake up?*”); the total daily minutes of sleep duration was calculated from these values. Sleep quality was assessed with a single item “*How well did you sleep last night?*” ranging from 1(*very badly*) to 7(*very well*).

## Results

### Analytical approach

The data were examined with regression and mediation analyses. We modeled the non-independence of the repeated measurement data by first determining the autoregressive structure of each time series using plots of their autocorrelation function and partial autocorrelation function (see Pollock et al., 2014 for a similar time series analysis of ideographic clinical symptoms). The functions indicated the presence of a second order autocorrelation in the time series (probably owing to the pairing of signals), so the second order lag of each dependent variable was included as a predictor in each model to control for its potential influence. For analyses examining associations with sleep variables, we aggregated sampled observations (e.g., individual instances of daydreaming) so that each day of the study was associated with one mean score per variable; we modeled the non-independence of daily data by including the first-order lag of the dependent variable in each regression model. This procedure allowed us to examine associations between sleep and average daily levels of daydreaming, dissociation, and mood. Alcohol consumption was controlled for in all analyses and all regressions were performed with bootstrapping (1000 samples).

### Response rate

All of the experience-sampling data were date and time stamped allowing us to check when the surveys were completed. Only the first answered survey was counted as a valid response if consecutively answered signals were less than 5 min apart. Of the 211 occasions on which experiences were reported, 81 (38%) were considered invalid; this left 130 observations upon which the following analyses were based which corresponds to a 54% valid response rate over the study period.

### Descriptive statistics

At the start of the study, the participant's average level of dissociative experiences according to the Dissociative Experiences Scale was 57.61 (measured on 100-point scale where >30 is considered a clinical cut-off for dissociation). Average values for each subscale of the DES also showed that symptoms of depersonalization/derealization (*M* = 73.33) and absorption (*M* = 75.67) were high; and levels of amnesia were relatively low (*M* = 29.83). Mirroring this, average levels for the Cambridge Depersonalization Scale showed high frequency and duration of dissociative symptoms (*M*_*frequency*_ = 4.03; *M*_*duration*_ = 4.45). Daydreaming was reported on 45% of sampled occasions. This frequency of daydreaming is within the range reported by other experience-sampling studies with non-clinical samples (e.g., 26%: Franklin et al., 2013; 30%: Kane et al., 2007; 47%: Killingsworth and Gilbert, 2010; 36%: Poerio et al., 2013; 60%: Song and Wang, 2012).

### Sleep duration and quality predicting daydreaming, dissociation, and mood

We examined whether sleep duration (*M* = 413 min; *SD* = 240 min) and quality (*M* = 2.00; *SD* = 1.54) independently predicted daily daydreaming and dissociative symptoms. Neither sleep duration nor quality predicted average daily daydreaming levels (duration: β = 0.005, *p* = 0.710, *B* = 0.00, *SE* = 0.00, 95%CI: 0.00, 0.00; quality: β = −0.002, *p* = 0.814, *B* = −0.001, *SE* = 0.00, 95%CI: −0.004, 0.005). However, average daily dissociative symptoms were negatively predicted by sleep quality (β = −0.17, *p* = 0.015, *B* = −0.03, *SE* = 0.01, 95%CI: −0.06, −0.00), but not sleep duration (β = −0.07, *p* = 0.399, *B* = 0.00, *SE* = 0.00, 95%CI: 0.00, 0.00). This suggests that although daydreaming incidence was not associated with sleep, dissociative symptoms were greater when sleep quality was poor. We also examined how sleep duration and quality were associated with average daily levels of anxiety and depression. Sleep duration did not predict either anxiety (β = −0.01, *p* = 0.961, *B* = 0.00, *SE* = 0.00, 95%CI: 0.00, 0.00) or depression (β = −0.07, *p* = 0.427, *B* = 0.00, *SE* = 0.00, 95%CI: 0.00, 0.00). Sleep quality was a negative predictor of depression (β = −0.17, *p* = 0.022, *B* = −0.05, *SE* = 0.00, 95%CI: −0.01, 0.00) but not of anxiety (β = −0.14, *p* = 0.115, *B* = −0.04, *SE* = 0.01, 95%CI: −0.10, 0.03).

### Daydreaming incidence predicting dissociation and mood

Next, we examined whether the occurrence of daydreaming predicted experiences of dissociation, anxiety, and depression. Daydreaming incidence was a significant negative predictor of dissociation (β = −0.28, *p* = 0.001, *B* = −0.25, *SE* = 0.06, 95%CI: −0.36, −0.13), anxiety (β = −0.43, *p* < 0.001, *B* = −0.73, *SE* = 0.13, 95%CI: −0.96, −0.46), and depression (β = −0.33, *p* < 0.001, *B* = −0.57, *SE* = 0.14, 95%CI: −0.83, −0.28) suggesting that experiences of dissociation, anxiety, and depression were more severe when daydreaming had occurred.

### Characteristics of daydreaming predicting dissociation and mood

Given the heterogeneity of daydreaming, we next examined whether the characteristics of daydreaming predicted experiences of dissociation, anxiety and depression. In contrast to our predictions, the fanciful nature of daydreams did not predict dissociation (β = −0.17, *p* = 0.175, *B* = −0.03, *SE* = 0.02, 95%CI: −0.07, 0.00), anxiety (β = −0.07, *p* = 0.229, *B* = −0.07, *SE* = 0.05, 95%CI: −0.17, 0.03), or depression (β = −0.22, *p* = 0.110, *B* = −0.10, *SE* = 0.05, 95%CI: −0.20, 0.02). However, the novelty of daydreaming was a significant negative predictor of dissociation (β = −0.37, *p* = 0.002, *B* = −0.06, *SE* = 0.02, 95%CI: −0.11, −0.03), anxiety (β = −0.51, *p* < 0.001, *B* = −0.18, *SE* = 0.04, 95%CI: −0.27, −0.10), and depression (β = −0.45, *p* < 0.001, *B* = −0.17, *SE* = 0.05, 95%CI: −0.28, −0.07). Likewise, the positivity of daydreams was a significant negative predictor of dissociation (β = −0.50, *p* < 0.001, *B* = −0.10, *SE* = 0.02, 95%CI: −0.14, −0.05), anxiety (β = −0.58, *p* < 0.001, *B* = −0.24, *SE* = 0.05, 95%CI: −0.33, −0.17), and depression (β = −0.63, *p* < 0.001, *B* = −0.28, *SE* = 0.06, 95%CI: −0.40, −0.17). These results suggest that repetitive and negative (but not fanciful) daydreams were associated with more severe experiences of dissociation, anxiety, and depression.

### Supplementary mediation analyses

Given the significant associations between daydreaming, mood, and dissociative symptoms, we were interested in further exploring whether mood mediated associations between daydreaming (incidence, novelty and emotional valence) and dissociation. To examine the role of mood as a potential mediator we ran a series of mediation analyses using PROCESS (Hayes, 2012) in which daydreaming variables were entered as the predictor variables, dissociation as the dependent variable, and anxiety and depression as the mediator variables. We entered the second order lag of the dependent variable and alcohol consumption as covariates in all models. The results of these mediation analyses are summarized in Table 1. Depression and anxiety significantly mediated relationships between: daydreaming incidence and dissociation and the novelty of daydreams and dissociation. Only anxiety was a significant mediator of the relations between the emotional valence of daydreams and dissociation.

**Table 1:** **Summary of mediation analyses**.

**Model**	**Path a**	**Path b**	**Path c (direct effect)**	**Indirect (mediated) effect**
Daydreaming Incidence-Anxiety-Dissociation	*B* = −0.73, *SE* = 0.13, *p* < 0.001	*B* = 0.26, *SE* = 0.04, *p* < 0.001	*B* = −0.04, *SE* = 0.07, *p* = 0.565	*B* = −0.19, *SE* = 0.05, 95%CI[−0.32, −0.10]
Daydreaming Incidence-Depression-Dissociation	*B* = −0.57, *SE* = 0.14, *p* = 0.001	*B* = 0.26, *SE* = 0.04, *p* < 0.001	*B* = −0.08, *SE* = 0.06, *p* = 0.218	*B* = −0.15, *SE* = 0.04, 95%CI[−0.26, −0.09]
Daydreaming Novelty-Anxiety-Dissociation	*B* = −0.19, *SE* = 0.04, *p* < 0.001	*B* = 0.18, *SE* = 0.06, *p* = 0.004	*B* = −0.03, *SE* = 0.02, *p* = 0.253	*B* = −0.04, *SE* = 0.02, 95%CI[−0.07, −0.01]
Daydreaming Novelty-Depression-Dissociation	*B* = −0.19, *SE* = 0.05, *p* < 0.001	*B* = 0.15, *SE* = 0.05, *p* = 0.009	*B* = −0.03, *SE* = 0.02, *p* = 0.132	*B* = −0.03, *SE* = 0.02, 95%CI[−0.06, 0.00]
Daydreaming Valence-Anxiety-Dissociation	*B* = −0.24, *SE* = 0.04, *p* < 0.001	*B* = 0.13, *SE* = 0.06, *p* = 0.032	*B* = −0.06, *SE* = 0.02, *p* = 0.019	*B* = −0.03, *SE* = 0.01, 95%CI[−0.06, −0.01]
Daydreaming Valence-Depression-Dissociation	*B* = −0.28, *SE* = 0.05, *p* < 0.001	*B* = 0.09, *SE* = 0.06, *p* = 0.117	*B* = −0.07, *SE* = 0.03, *p* = 0.017	*B* = −0.03, *SE* = 0.02, 95%CI[−0.06, 0.01]

*B, Unstandardized path coefficients, SE, Standard error. Ninety-five percent Confidence intervals for indirect effects are based on 1000 bootstrapped samples. Path a refers to the effect of the predictor on the proposed mediator (e.g., daydreaming on anxiety), Path b refers to the effect of the mediator on the dependent variable (e.g., anxiety on dissociation), Path c refers to the direct effect of the predictor on the dependent variable considering the mediator. The indirect effect provides an indication of statistical mediation such that 95%CIs excluding zero are considered statistically significant at the p < 0.05 level. Alcohol consumption and the second order lag of the dependent variable were entered as covariates in all models*.

## Discussion

**Figure 2:**
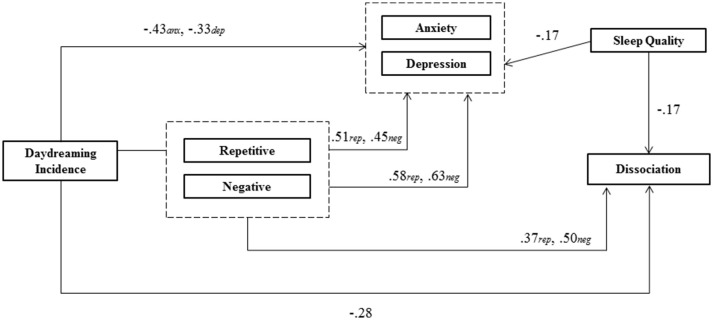
**An updated model of how daydreaming is related to dissociation**. Values represent regression coefficients for analyses in the current study.

In this intensive clinical case study we used experience-sampling methodology to sample sleep, daydreaming (and its characteristics), mood, and dissociative symptoms in an individual meeting diagnostic criteria for depersonalization/derealization disorder (DDD; American Psychiatric Association, 2013). Based on previous research and theory on both the role of sleep and “dream-like” intrusions in the maintenance of dissociation and the potential role of daydreaming in this process, we proposed an initial model (Figure 1) explaining how sleep and daydreaming are linked with mood and dissociative symptoms. The evidence for this model based on the results of the current study are as follows (an updated model based on the present study is presented in Figure 2):

First, although we expected sleep disturbances to be associated with greater daydreaming we did not find evidence to suggest that either sleep duration or quality predicted average daily incidence of daydreaming. This finding is unexpected because a range of previous evidence on sleep and daydreaming suggests that sleep disruptions are associated with greater daydreaming frequency (e.g., Kunzendorf et al., 1983; Mikulincer et al., 1990; Carciofo et al., 2014; Denis and Poerio, 2016). We suspect that this null finding may be because the method examining daydreaming incidence (i.e., averaging whether the participant was daydreaming or not across the number of questionnaires answered each day) was unable to accurately characterize daily daydreaming rates. On average, the participant answered 3.7 questionnaires each day (modes = 1, 3), which may not have provided an accurate assessment of the likelihood of daydreaming occurrence. Notably, previous research linking sleep experiences to daydreaming frequency has involved estimates of thoughts over during a brief time period (i.e., a laboratory task; Mikulincer et al., 1990) or has been based on retrospective/global judgments of daydreaming frequency (Carciofo et al., 2014). To appropriately characterize daydreaming incidence with experience-sampling methodology, future research would benefit from more frequent sampling and/or a retrospective daily evaluation of daydreaming frequency. Firmly establishing the link between sleep disturbance and daydreaming in dissociative disorder is important in order to provide empirical evidence to support theoretical ideas that sleep disruptions are associated with an increase of “dream-like” intrusions into waking life (van der Kloet et al., 2012b) often reported in dissociative disorders (American Psychiatric Association, 2013).

Second, poorer self-reported sleep quality (but not sleep duration) was associated with significantly greater severity of dissociative symptoms across the following day. This finding is consistent with research and theory highlighting the importance of sleep disturbance in dissociation (e.g., Watson, 2001; Levin and Fireman, 2002; Agargun et al., 2003; Giesbrecht and Merckelbach, 2004; Giesbrecht et al., 2007; Koffel and Watson, 2009a; van der Kloet et al., 2012b; van Heugten–van der Kloet et al., 2015).

Our study not only confirms this association but it is also the first to provide evidence for a positive association between disrupted sleep and dissociative symptoms using an intensive repeated measures design of a dissociative disorder. Measuring fluctuations in sleep quality over time and assessing accompanying dissociative symptoms “in the moment” overcomes the potential biases involved in using cross-sectional research based on retrospective/global reports (e.g., Bradburn et al., 1987). Our research suggests that the relationship between sleep and dissociation in dissociative disorders over time is important and that poor quality sleep may be a factor that might maintain or exacerbate dissociative symptoms. The finding that sleep quality but not duration was associated with dissociation is of particular interest because previous research has suggested that whereas mood disorders appear to be linked with insomnia, dissociation appears to be uniquely linked to unusual sleep experiences (Koffel and Watson, 2009b; van Heugten–van der Kloet et al., 2014). Our research broadly supports this finding to the extent that subjective sleep quality is a measure that reflects or captures sleep disturbances. Having established the link between sleep quality and dissociative symptoms in an intensive longitudinal design, future research would benefit from more extensive measurement of sleep disturbance across both subjective (e.g., additional measures on the presence/absence of unusual sleep experiences such as nightmares and hallucinations; and dreaming content) and objective measures (e.g., actigraphy). The use of more objective measures of sleep and/or more sensitive self-report measures (e.g., the Pittsburgh Sleep Quality Index; Buysse et al., 1989) would be particularly important in future research because the present study is limited by the use of single items to measure sleep quality and duration.

Third, daydreaming and its characteristics were associated with both mood and dissociative symptoms. In line with our predictions, repetitive and negative daydreams were associated with greater feelings anxiety, depression, and dissociation. This is consistent with previous research on the role of repetitive thought and daydreaming in clinical disorders (e.g., Segerstrom et al., 2003; Watkins, 2008). However, our study extends these ideas beyond depression and anxiety to dissociation, indicating that daydreaming incidence and content are important factors to assess in dissociative disorders. Notably, these findings support theoretical (but until now empirically untested) ideas that dream-like intrusions in daily life are involved in the proximal development and progression of dissociative disorders (e.g., van der Kloet et al., 2012b). Not only was the occurrence of daydreaming associated with dissociative symptoms, but the extent to which daydreaming negatively impacted on mood and dissociation also depended on the characteristics of daydreams. This highlights the need to consider the content (rather than just the occurrence) of daydreaming in clinical disorder and supports a growing body of research showing that in order to determine the positive and/or negative impact of daydreaming on well-being it is vital to measure the heterogeneity of the experience (e.g., Mar et al., 2012; Franklin et al., 2013; Ottaviani and Couyoumdjian, 2013; Poerio et al., 2013, 2015a,b, 2016; Ruby et al., 2013; Smallwood and Andrews-Hanna, 2013).

Fourth, supplementary mediation analyses examined the possibility that mood might mediate the association between daydreaming and its characteristics on dissociation. We found evidence that, in general, feelings of anxiety and depression mediated the positive statistical effects of repetitive and negative daydreaming on dissociative symptoms. This suggests that at least part of the reason why daydreaming is associated with dissociative symptoms arises indirectly from the effect of daydreaming on mood. This is consistent with previous research, which has consistently documented the strong and important impact of various types of imagery on mood in emotional disorders (Holmes and Mathews, 2010) and, in non-clinical samples, the well-established link between daydreaming content and later mood states (e.g., Franklin et al., 2013; Poerio et al., 2013; Ruby et al., 2013). Examining the role of mood in dissociative disorders is important because anxiety and depression are associated with the severity of dissociative symptoms in clinical populations and a previous diagnosis of depression and/or anxiety has been identified as the main risk factor for depersonalization disorder (Baker et al., 2003). This, combined with the present findings, suggests that research examining the development and progression of dissociative disorders would benefit from exploring interactions and the causal relationships between daydreaming, mood, and dissociative symptoms.

One additional and unexpected finding deserves particular mention. We predicted that fanciful daydreaming would be associated with worse mood and greater dissociative symptoms. Not only were the relationships between fanciful daydreaming and feelings of anxiety, depression, and dissociation non-significant but the direction of the relationship between fanciful daydreaming and dissociation was also the opposite of what would be predicted based on previous research and theory. Previous research has consistently identified fantasy-proneness as a correlate of dissociative disorders (Rauschenberger and Lynn, 1995; Giesbrecht and Merckelbach, 2006) and excessive involvement in fantasy is proposed to be a symptom of dissociation (van der Kloet et al., 2012b). To our knowledge, this study is the first to measure fanciful thought as it occurs momentarily in daily life and has failed to find evidence of a link between fanciful mentation and dissociative symptoms. Although, any inferences based on this finding must be considered tentative given our *N* = 1 sample, we suggest that the often-cited link between fantasy and dissociation should be reconsidered and examined in relation to actual daily experiences of fantasy, rather than composite and global tendencies of imaginative involvement (see also Cima et al., 2001; Bremner, 2010). Indeed, it has previously been suggested that the association between fantasy-proneness and dissociation may arise simply because measures assessing both constructs show substantial overlap in item wording (Klinger et al., 2009).

Despite the unique contributions stemming from the present study, it is important to highlight that (a) our results may not be applicable to all individuals with dissociative disorders because this is a single case study specific to DDD and (b) the causal nature of these results should not be overstated. The correlational nature of the study design and the use of self-report measures make it important to emphasize that this study cannot shed light on the causal or temporal nature of the observed associations. For example, it is possible that the participant's own theories about his disorder (of which we have no knowledge) may have affected his reporting (e.g., that negative daydreams should be associated with worse mood and dissociation). It is also likely that the associations observed are bi-directional. For example, although we examined how daydreaming was related to mood because daydreaming was measured as occurring before the signal (or in the preceding 5 min) whereas mood was measured “right now,” previous research has shown that prior mood also influences the frequency and nature of daydreaming. Research has revealed that a negative mood (particularly sadness) is associated with increased daydreaming in both laboratory settings (Smallwood et al., 2009) and in daily life (Poerio et al., 2013). This suggests that rather than (or in addition to) daydreaming predicting negative “in the moment” feelings, daydreaming is also preceded or caused by negative mood. Although, we failed to find associations between sleep and daydreaming incidence, other research has suggested that this relationship is also bi-directional (i.e., that daydreaming is linked with subsequent difficulty falling asleep; Ottaviani and Couyoumdjian, 2013). Likewise, dissociation may be a predictor as well as a consequence of daydreaming incidence and content. For example, the daydreaming state may exacerbate symptoms in someone who is prone to dissociation, while states of dissociation could make daydreaming more likely (e.g., dissociation may be linked with the inability to inhibit task-irrelevant thoughts due to a lack of metacognitive control or distractibility; Guralnik et al., 2007). Although, this previous research might be suggestive of alternative explanations to the current results, we suspect that these directions of influence are not mutually exclusive or contradictory. Indeed, considering the bi-directional associations between sleep, daydreaming, mood, and dissociation is likely to represent a more comprehensive account of the cyclical and dynamic nature of moment-to-moment cognition and emotion, particularly within clinical disorders (Borsboom and Cramer, 2013). Future work would profit from examining these variables with more advanced and intensive experience-sampling designs involving multiple participants and in response to intervention. This would enable an examination of the relative strength of different directions of influence (e.g., is the effect of sleep on dissociation stronger than the effect of dissociation on sleep?) and of individual differences that moderate the relationships.

We have argued and provided evidence for the idea that daydreaming is an important state of consciousness relevant to dissociative disorders. However, an important outstanding question involves the precise role of daydreaming in dissociative disorders. Daydreaming has sometimes been conceptualized as a cognitive failure or attention lapse (e.g., Cheyne et al., 2006; McVay and Kane, 2010), or as engagement with fantasy and imagery (e.g., Oettingen and Mayer, 2002), and more recently as an important state of cognition for psychosocial functioning (e.g., Poerio and Smallwood, Submitted). The former two conceptions of daydreaming are of direct relevance to dissociative disorders, with research suggesting that clinical dissociation is linked both with cognitive failures and distractibility (Giesbrecht et al., 2008) and with a preoccupation and absorption with fantasy (Segal and Lynn, 1993). Although, our findings suggests that the fanciful nature of daydreaming may not be as pertinent to the maintenance of dissociative symptoms as previously suggested (at least in this particular individual), future work should further explore relevant characteristics of daydreaming to investigate whether its relationship with dissociation arises from cognitive failure or fantasy immersion. Future work might also examine the less explored aspects of daydreaming and psychosocial functions in relation to dissociation; by, for example, examining how aspects of social cognition (e.g., the ability to distinguish between self and other) during daydreaming are linked with symptoms of dissociation (in particular depersonalization).

There are several potential aspects of daydreaming that might shed light on this issue of whether daydreaming in dissociation reflects cognitive failure or fantasy proneness—taking into account both the nature of daydreaming and the context in which it occurs. First, drawing on methods used to examine daydreaming in laboratory settings (e.g., Konishi et al., 2015), research could investigate whether rates of daydreaming during tasks requiring attention are related to the severity of dissociative symptoms (e.g., as measured by the DES or by comparing clinical and non-clinical samples). Second, experience-sampling research could investigate how the extent of immersion or absorption in daydreams is related to dissociative symptoms with the expectation that these dimensions of daydreaming would be associated with, and possibly exacerbate, dissociative symptoms. Although, daydreaming is a ubiquitous experience, it is possible that daydreaming in dissociation is more “immersive” and so individuals with dissociative disorders struggle to disengage from daydreams and ground themselves in reality. Third and relatedly, an important dimension to be explored would be the controllability and intentionality of daydreaming. Recent research has linked spontaneous (rather than deliberate) daydreaming with clinical disorders (Marchetti et al., 2016) suggesting that they may be more detrimental to mental health. This prediction fits well with the idea that uncontrollable and spontaneous daydreams may be more detrimental to dissociation because they are further toward the dreaming end of the wake-sleep cognition continuum. Indeed, this is mirrored by associations observed in sleeping cognition because uncontrollable sleep disturbances (e.g., nightmares) are typically more strongly associated with dissociative experiences than controllable sleep mentation such as lucid dreaming (Koffel and Watson, 2009a).

Finally our findings motivate the intriguing possibility that sleep and daydreaming are potential intervention targets for dissociative disorders. Research has already demonstrated that improving sleep quality through a sleep hygiene intervention can reduce dissociation (van der Kloet et al., 2012a). Although, the potential for intervention requires future work, we believe that examining the state of daydreaming and its characteristics in dissociative disorders will enrich our understanding of how dissociative symptoms evolve and are potentiated as they occur in daily life. There is scope for targeted interventions aimed at changing the negative aspects of daydreaming whilst maintaining its functional outcomes (e.g., planning, creativity, and social well-being). In terms of the clinical methods to help to change negative aspects of daydreams, use of imagery re-scripting (i.e., actively manipulating negative daydreaming imagery) holds some promise (Wild and Clark, 2011). For more complex patient problems, sleep hygiene, and daydream content interventions could occur in the initial phase of treatment, so that dissociation is reduced and the patient is stabilized, to enable them to engage more effectively in psychotherapeutic work on past trauma. In keeping with the hourglass model of evaluation (Salkovskis, 1995), further *N* = 1 outcome studies offer the opportunity to study, in detail, responsivity to suggested phases of treatment, before proceeding onto larger group studies. In conclusion, this research has shed new light on the relationships between sleep, daydreaming, mood, and dissociation in DDD and highlights exciting avenues for future clinical and research work.

## Author contributions

All authors conceived of, and designed, the study. GP collected and analyzed the data with assistance and contributions from SK, PT. GP drafted the manuscript; SK, PT provided revisions. All authors read and approved the final manuscript.

## Funding

This research was supported by the Economic and Social Research Council [grant number ES-J500215-1] awarded to GP whilst at the University of Sheffield. GP is now supported by the European Research Council [grant number: 646927].

### Conflict of interest statement

The authors declare that the research was conducted in the absence of any commercial or financial relationships that could be construed as a potential conflict of interest.
